# Multi-Class Classification of Medical Data Based on Neural Network Pruning and Information-Entropy Measures

**DOI:** 10.3390/e24020196

**Published:** 2022-01-27

**Authors:** Máximo Eduardo Sánchez-Gutiérrez, Pedro Pablo González-Pérez

**Affiliations:** 1 Colegio de Ciencia y Tecnología, Universidad Autónoma de la Ciudad de México, Ciudad de Mexico 06720, Mexico; maximo.sanchez@uacm.edu.mx; 2 Departamento de Matemáticas Aplicadas y Sistemas, Universidad Autónoma Metropolitana-Cuajimalpa, Ciudad de Mexico 05348, Mexico

**Keywords:** medical data and signals, machine learning, restricted Boltzmann machine, feature selection, discriminant pruning, information-entropy measures

## Abstract

Medical data includes clinical trials and clinical data such as patient-generated health data, laboratory results, medical imaging, and different signals coming from continuous health monitoring. Some commonly used data analysis techniques are text mining, big data analytics, and data mining. These techniques can be used for classification, clustering, and machine learning tasks. Machine learning could be described as an automatic learning process derived from concepts and knowledge without deliberate system coding. However, finding a suitable machine learning architecture for a specific task is still an open problem. In this work, we propose a machine learning model for the multi-class classification of medical data. This model is comprised of two components—a restricted Boltzmann machine and a classifier system. It uses a discriminant pruning method to select the most salient neurons in the hidden layer of the neural network, which implicitly leads to a selection of features for the input patterns that feed the classifier system. This study aims to investigate whether information-entropy measures may provide evidence for guiding discriminative pruning in a neural network for medical data processing, particularly cancer research, by using three cancer databases: Breast Cancer, Cervical Cancer, and Primary Tumour. Our proposal aimed to investigate the post-training neuronal pruning methodology using dissimilarity measures inspired by the information-entropy theory; the results obtained after pruning the neural network were favourable. Specifically, for the Breast Cancer dataset, the reported results indicate a 10.68% error rate, while our error rates range from 10% to 15%; for the Cervical Cancer dataset, the reported best error rate is 31%, while our proposal error rates are in the range of 4% to 6%; lastly, for the Primary Tumour dataset, the reported error rate is 20.35%, and our best error rate is 31%.

## 1. Introduction

Medical data commonly refers to the vast amount of information collected and stored from patients each time they attend a medical consultation or remain hospitalised. A significant portion of this information corresponds to data involving numbers related to the patient’s health. These records are not only helpful to obtain traces from the patient’s medical history, but they are also useful for research purposes. Medical data can include [1]: (a) numerical data corresponding to the main vital signs, such as pulse rate, body temperature, respiration rate, and blood pressure; (b) diagnostic information, such as identification of the disease, syndrome, disease entity, or pathology, among others; (c) treatment information, e.g., medication or therapy to be followed by the patient; (d) disease or patient registries; and (e) clinical trial data, i.e., clinical information that has been gathered thanks to experiments associated with clinical research. Due to a large number of medical datasets and to the myriad of categories each case may fall into, it becomes increasingly challenging to process and analyse data samples, such as attempting to identify data patterns across different patients suffering from the same medical conditions such as chronic diseases, treatment responsiveness, or the leading causes of death. For this reason, medical science has started to utilise technology to produce reliable medical datasets to improve medical data analysis.

Physicians assess the characteristics of patients (e.g., clinical picture) in an attempt to comprehend their health and diseases. At the same time, computer sciences integrate these into models with the results of these measurements, tests, and observations. At present, the use of data mining and machine learning techniques for medicine has accelerated in growth, focusing on the health of the patient and the ability to predict diseases. Some benefits of medical data analysis are: (a) its patient-centred and structured information, (b) the ability to cluster the population into groups according to features such as diagnosis or disease stage, (c) the ability to carry out analyses of drug effectiveness and effects in people, and (d) clinical patterns [2,3]. Novel information technologies and computational methods can be used to improve the analysis and processing of medical data. These approaches are helpful for medical data analysis since several medical datasets—such as those on disease characterisation—could be analysed through different methods, such as machine learning, data science, or predictive analytics [4].

Medical data analysis is essential for cancer research to combine the information reported in the cancer literature, cancer databases, and cancer medical records. All this information allows the identification of specific patterns that characterise the different types and stages of cancer. The processing and analysis of these patterns based on data mining and machine learning techniques should help in the timely detection of this disease. In essence, machine learning could be described as an automatic learning process derived from concepts and knowledge without deliberate system coding. Achieving this learning requires observing previously seen data patterns that contribute to adjusting a model capable of producing better results.

In this work, we propose a machine learning model for the multi-class classification of medical data. The model, which is composed of two components, a restricted Boltzmann machine and a classifier system, uses a discriminant pruning method to select the most salient neurons in the hidden layer of the neural network, which implicitly leads to a selection of features for the input patterns that feed the classifier system. Our study aims to investigate whether information-entropy measures may provide evidence for discovering useful neurons in a neural network for medical data analysis, particularly cancer research, by using three cancer databases: Breast Cancer, Cervical Cancer, and Primary Tumour. Our proposal aims to investigate the post-training neuronal pruning methodology using dissimilarity measures inspired in the information-entropy theory. In a previous paper [5], we presented the conceptual framework for discriminative pruning based on statistical distances to calculate each RBM’s hidden neuron’s utility in a classification problem. Here, we broaden the approach to classifying multiple classes within the study of medical data characterising various cancer forms.

This work’s methodological approach applies machine learning techniques, such as neural network pruning, restricted Boltzmann machines, k–nearest neighbours, and information-entropy measures. All of these are introduced in Section 2, including the description of the cancer datasets. Section 3 discusses the achieved results. Finally, Section 4 presents our findings and future potential research.

The main contributions of this work can be summarized in terms of (1) the implementation of a ranking strategy for the pruning process of dense neural networks, (2) the search for the most robust information-entropy measures to rank and prune an artificial neural network, and (3) the attainment of favourable classification rates with savings of 60 to 95 per cent of hidden neurons.

To better comprehend the terminology used in the following sections, a brief definition of the different notations and terms is provided in Table 1.

## 2. Material And Methods

### 2.1. Machine Learning Techniques

One of the fundamental tools of machine learning is artificial neural networks (ANNs); the training efficiency of these ANNs is improved when large volumes of data are presented as input. This process consists of two phases: training and inference. The training process involves labelling large volumes of data, while the inference step makes conclusions and labels new data using prior information. In neural network models, the processing unit is the artificial neuron, which takes multiple input signals integrating them with a vector of weights to produce the output. These artificial neurons are commonly non-linear processing units whose role is to convert and extract features.

**Figure 1:**
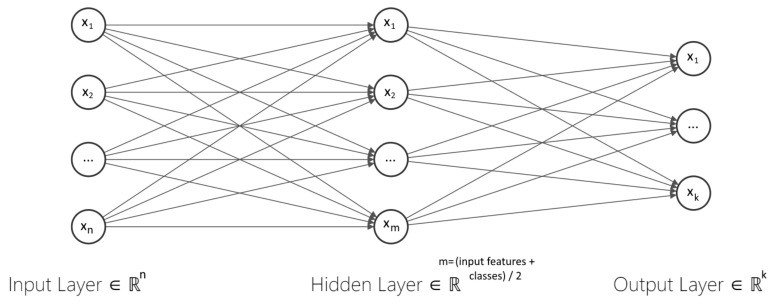
Generic multi–layer perceptron model. The input layer corresponds to the dimension of the input vectors. The hidden layer contains (# input features + # classes)/2 neurons. Finally, the number of neurons in the output layer corresponds to the same number of classes.

An ANN can be implemented as a classifier since it finds the most appropriate boundary between two or more classes. Hence, it may discern the structural differences between two or more given classes, identify the space that separates each one, and determine the likelihood of a given data point belonging to a particular class. An ANN connects multiple neurons or perceptrons partitioned into the input, hidden, and output layers. The neurons compose a directed acyclic graph, meaning that the paths connect nodes in layers from one layer to the next, as shown in Figure 1. Each neuron, excluding the input ones, has an activation function, a bias, and connecting weights, which the ANN trains by backpropagation in a supervised learning fashion [6] so that the error value can be updated in a much more successful way.

**Figure 2:**
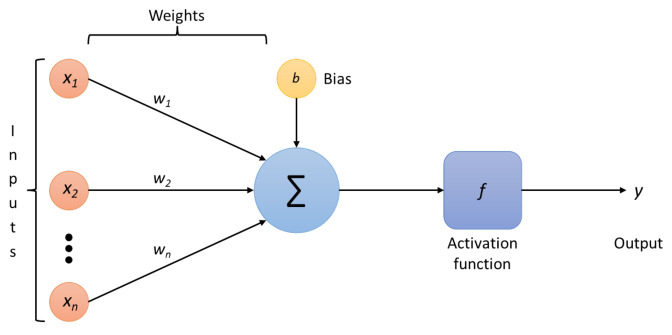
Operations done by a neuron.

The operations done by each neuron are straightforward. First, it adds up the value of every neuron from the previous column it is connected to. In Figure 2, the $\left(x_{1},x_{2},x_{n}\right)$ inputs are coming to the neuron, so *n* neurons of the previous column are connected to the neuron. This value is multiplied, before being added, by its weights $\left(w_{1},w_{2},w_{n}\right)$, which determines the connection between the two neurons. Each neuron’s connection has its weight, which are the only values modified during the learning process. Moreover, a bias value may be added to the total value calculated. It is not a value coming from a specific neuron that allows the model to move if needed to fit the data. After those operations, the neuron finally applies an activation function to obtain the output value.

In Figure 2, the full expression of the Σ symbol is given in expression (1), where j=0 is the bias neuron:

(1) $$\Sigma =\sum_{j=0}^{n}w_{j}x_{j}$$

**Figure 3:**
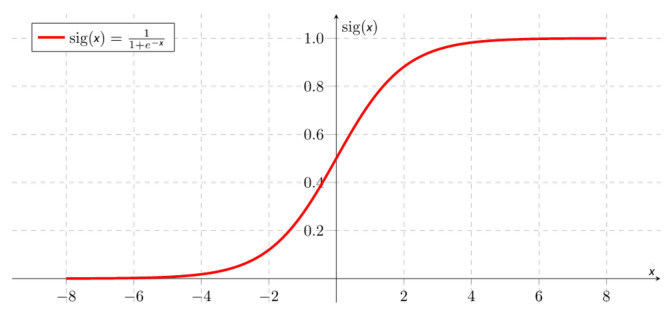
Sigmoid function.

The activation function usually helps to turn the total value calculated before to a number between 0 and 1 (for example, the sigmoid function shown in Figure 3 and expression (2)). In summary, a neuron takes all the values from weight-connected neurons multiplied by their respective weight, adds them, and applies an activation function. Then, the neuron propagates its output value to other neurons. In the end, the values in the last layer are used to determine the class output.

(2) $$f=sig\left(x\right)=\frac{1}{1+e^{-x}}$$

Artificial neural networks transform their internal attributes by taking abstractions of the preceding layer. This procedure is commonly applied in many areas that deal with mass data generation, such as the analysis of large volumes of medical and clinical data, analysis and document recognition, object detection, voice recognition, image classification, detection of pedestrians in road environments, natural language processing, and voice activity detection [7].

An important and emerging area of research in artificial neural networks is related to optimising their parameters. The heuristic nature in which neural networks are constructed is one of their major limitations. Therefore, choosing an appropriate network topology for a given problem is a difficult task that remains an emerging field with much to explore.

Recent applications of neural networks commonly use large data sets produced by an immense range of applications and devices in which we are immersed. Machine learning algorithms, combined with massive data sets, increase the need to optimise computing resources since, as the data size increases, the required processing time also increases. Therefore, there is a need to investigate efficient ways to deal with network topologies.

### 2.2. Pruning Techniques for Machine Learning Models

A few proposals have been suggested for structuring effective and shallow networks that offer better performance. A common approach to determine the size of the network is through the use of heuristics, generally seeking a balance between performance and generalisation capability in a validation set. Another approach considers a minimal neural network topology and ‘grows’ it until a satisfactory performance is achieved. Another technique uses ‘pruning’ methods.

Pruning is a method used to decrease the size of a neural network to make it more efficient in terms of resources such as execution time, memory usage, energy, and the number of computations required [8]. During pruning, the neurons themselves are removed in an iterative process, where more pruning is performed as long as the performance (in terms of the network’s accuracy) remains high. The main reason for using pruning is to make a neural network more efficient. Nevertheless, extensive pruning generally decreases the accuracy of the classifier.

Pruning methods typically start via preparing a neural network that is sufficiently large to accomplish good performance. Then, according to specific criteria, the neurons are deleted from the neural model to retrain the network. This approach is typically rehashed until some convergence condition is met. If the condition is not satisfied, the network with the lowest error rate is selected. This form of pruning is known as PTP (post-training pruning). This method selects the neurons that contribute the most and prunes the ones that contribute the less.

### 2.3. Information-Entropy Measures for Discriminative Pruning

The proposed methods for measuring the degree of neuronal discrimination in a multiclass classification problem were born out of the need to determine if the neurons in a neural network learned the underlying class boundaries and their contribution to the classification task. To this end, our proposal uses these information-entropy measures to rank the neurons in the RBM hidden layer according to their output values and then prunes the less salient ones according to this rating.

#### 2.3.1. Fisher Score

This metric evaluates distances in different feature-subset spaces between each data point so that intraclass distances are as small as possible while interclass distances are as far apart as possible. The Fisher score will calculate the outcome for the *i*-th feature $S_{i}$ as:

(3) $$S_{i}=\frac{\sum_{j=1}^{J}n_{j}{\left(\mu_{ij}-\mu_{i}\right)}^{2}}{\sum_{j=1}^{J}n_{j}\rho_{ij}^{2}}$$

where $\mu_{i}$ is the mean of the *i*-th feature, $\mu_{ij}$ is the mean of the *i*-th feature in the *j*-th class, $\rho_{ij}$ is the variance of the *i*-th feature in the *j*-th class, and $n_{j}$ is the number of cases in the *j*-th class.

#### 2.3.2. Pearson’s Correlation Coefficient

Pearson’s correlation coefficient (PCC) measures the statistical relationship between two continuous variables (features). The results’ values are in the range of [−1,1]; this gives information about the magnitude of the correlation, as well as the direction of the relationship. The PPC is given by expression (4):

(4) $$r=r_{xy}=\frac{\sum x_{i}y_{i}-n\bar{x}\bar{y}}{\left(n-1\right)\sigma_{x}\sigma_{y}}$$

where σ denotes the standard deviation of the sample. The linear correlation coefficients between observations are expressed as an *n*-by-*n* pairwise matrix.

#### 2.3.3. ANOVA

The one-way analysis of variance is used to assess if data belonging to several sets have a similar mean and to identify whether the mean of two or more datasets are statistically different. ANOVA is used to test the null hypothesis:

(5) $$H_{0}=\mu_{1}=\mu_{2}=\mu_{3}=\ldots =\mu_{j}$$

where J= number of groups and μ= group mean.

#### 2.3.4. Information Gain

Information gain (IG) is calculated by the feature’s contribution to reducing the total entropy by using how much variance the data has as a criterion for feature quality. Given a random variable (feature) ***X***, its entropy ***H*** is given by:

(6) $$H\left(X\right)=-\sum_{x}p\left(x\right)\log_{2}p\left(x\right)$$

The conditional entropy H(X/Y) is defined by Equation (7).

(7) $$H\left(X|Y\right)=\sum_{x,y}p\left(x,y\right)\log_{2}p\left(x|y\right)$$

where *X* is a random variable (feature), *Y* is an observer random variable (feature), and p(x,y) is the joint probability.

Information gain (IG) depicts how much information the observed feature *Y* provides about the feature *X*, that is, how much the entropy of *X* decreases from the information provided by *Y*:

(8) IG=H(X)−H(X|Y)

#### 2.3.5. Gain Ratio

The gain ratio measures the amount of information provided by a feature about a class by normalising information gain (8). It also measures the reduction in entropy. A value of 1 indicates that feature *X* completely predicts feature *Y*, and a value of 0 denotes that the *Y* and *X* features are uncorrelated.

The gain ratio is given by:

(9) $$GR=\frac{H(X)-H(X|Y)}{H(X)}=\frac{IG}{H(X)}$$

#### 2.3.6. Symmetric Uncertainty

Symmetric uncertainty (SU) evaluates the worth of features symmetrically by normalising IG by the sum of the features’ entropy’s (*X* and *Y*). Therefore, SU(X,Y) is the same as SU(Y,X):

(10) $$SU=2\frac{H(X)-H(X|Y)}{H(X)+H(Y)}=2\frac{IG}{H(X)+H(Y)}$$

where *X* and *Y* represent two independent features.

#### 2.3.7. ReliefF

ReliefF is not a metric, but an algorithm [9]. The ReliefF algorithm estimates the quality of features based on how well the feature can differentiate between similar instances. The key factor is that the ReliefF algorithm does not assume the conditional independence of the features.

As can be seen in expression (11), $W_{f}$ is the weight assigned to each feature considering its role to discriminate between classes:

(11) $$W_{f}=a-b$$

where a = p(different feature value | different class), and b = p(different feature value | same class).

#### 2.3.8. One–R

One–R is not a metric but an algorithm based on the classes’ frequency table to select the best feature or predictor (neuron in this case) from the input vector [10]. It generates one rule for each value of each feature and then selects the one with the smallest error (see Algorithm 1).
**Algorithm 1** One rule [10].**for each** feature *f*    **for each** instance *i* of *f*        Count the number of times each *i* occurs in the target class        Choose the most occurring class *c*        Build a rule that assigns *c* to *i*    **end for**    Determine the cumulative error of each feature’s rule**end for**Select the feature with the lowest cumulative error

### 2.4. The Restricted Boltzmann Machine

The restricted Boltzmann machine (RBM) is a neural network that models a non-directed and symmetrical connection between the visible and hidden nodes without any intra-layer connection. A RBM learns a probability distribution when a set of input patterns is offered to the network. On the other hand, a deep belief network (DBN) is a deep neural network based on many hidden unit layers. In a DBN, each pair of connected layers is a RBM. The input layer establishes the input of the data, while the hidden layer characterises this input’s abstract description.

The units in a restricted Boltzmann machine are connected across layers, but there is no intra-layer communication; this restriction is what makes a restricted Boltzmann machine. This lack of intra-layer connections allows the weights of the connections between the visible and hidden nodes to be learned by Hinton’s contrastive divergence algorithm [11]. In conjunction with deep belief networks, these neural networks have been applied to address several problems, for example, natural language processing [12], image classification [13], forecasting time series [14], and voice synthesis [15], among others.

In an RBM, an energy function given by expression (12) is defined for each visible-hidden arrangement of Gaussian neurons.

(12) $$E\left(v,h\right)=\sum_{i}\frac{{\left(v_{i}-a_{i}\right)}^{2}}{2\sigma_{i}^{2}}-\sum_{i}\sum_{j}\frac{v_{i}}{\sigma_{i}^{2}}h_{j}w_{ij}-b^{\prime }h$$

where *w* is the visible-hidden weights matrix, and *a*, and *b* are the respective bias vectors.

For a given RBM configuration, to calculate the joint probability p(v,h), the expression (13) is used:

(13) $$p\left(v,h\right)=\frac{\exp^{-E(v,h)}}{Z}$$

where the partition function *Z* is given by expression (14).

(14) $$Z=\sum_{v,h}\exp^{-E(v,h)}$$

The RBM assigns to the vector *v* the probability given by expression (15).

(15) $$p\left(v\right)=\frac{1}{Z}\sum_{h}\exp^{-E(v,h)}$$

The conditional independence in a RBM comes from the lack of intra-layer connections. This independence allows us to write the conditional probabilities as in expression (16).

(16) $$p\left(h_{j}=1|v\right)=\sigma (\sum_{i}\frac{v_{i}}{\sigma_{i}^{2}}w_{ij}+b_{j})$$

where

(17) $$\sigma \left(x\right)=\frac{1}{1+\exp^{-x}}$$

and

(18) $$p\left(v_{i}=v|h\right)=N\left(v|\sum_{j}h_{j}w_{ij}+a_{i},\sigma_{i}^{2}\right)$$

with mean μ and variance $\sigma^{2}$. $N(\cdot |\mu ,\sigma^{2})$ expresses the Gaussian probability density.

In [16], a RBM training guide is given. The algorithm applies contrastive divergence (CD) to find the weights and biases, and in turn, the CD uses the Gibbs sampling method to update the RBM parameters.

Regarding the recognition, classification, and clustering of medical data, deep learning models—RBMs, DBNs, and other models—have been used successfully and have demonstrated superior performance in solving many medical data processing problems compared with other machine learning techniques [17,18].

**Figure 4:**
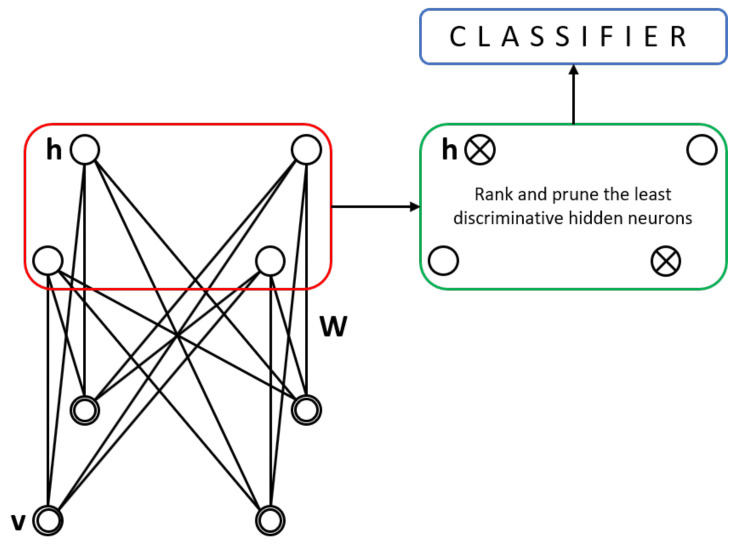
Discriminative pruning of hidden neurons. The process includes a classifier that takes the outputs of the most discriminative neurons from an RBM.

Recently, RBMs have been used as a mechanism for the initialisation of weights in artificial neural networks or as feature extractors [19,20]. As Figure 4 shows, a RBM exhibits one outer layer (input) and one hidden layer; unlike the traditional multilayer perceptron, there is no third output layer. However, the neurons are connected as in an MLP; there are inter-layer but not intra-layer connections. The probability distribution of the data can be learned thanks to the stochastic nature of the RBM.

The hidden layer outputs can be fed to several classifiers after the RBM has been trained. In this step, where the hidden layer’s output serves as input feature vectors to some classifier, we can evaluate their discriminative capacity. This process resembles a feature selection process. We analyse several statistical distances to rank the hidden layer units according to their discriminative power to remove (prune) the less discriminative ones, and then analyse the resulting performance to prove its statistical significance.

The advantages of pruning a bigger network were discussed in a recent article [5]. The question arises as to whether the solution to an audio signal multi-class classification task is still helpful to medical data and medical data analytics. The algorithm, as described in [5], requires a RBM trained in an unsupervised fashion to obtain the propagated values for each training sample from the different classes in the dataset. The outputs of each hidden neuron are then used to calculate their discriminative value. Each neuron is ranked accordingly to those discriminative values.

### 2.5. The K-Nearest Neighbours Algorithm

The k-NN classification algorithm is often referred to as memory-based learning or instance-based learning. These terms indicate the central concept of k-NN: creating a model from the training data set and then using this data to classify new observations. The k-NN algorithm (Algorithm 2) uses a majority voting mechanism. k-NN collects data from a training set and uses it to classify the new examples. For each new example, the k≥1 closest neighbours in the training dataset are evaluated to assign the new example to the class, which is decided by voting on the nearest neighbours. By changing the value of k, the balance between generalisation and overfitting can be guided.
**Algorithm 2** Discriminative evaluation.**Input:**   $D=\left(x_{1},c_{1}\right),\ldots ,\left(x_{N},c_{N}\right)$ $x=(x_{1},\ldots ,x_{n})$ a new example to be classified  1:**for each** example of the training set $\left(x_{i},c_{i}\right)$2:    Compute $d_{i}=d\left(x_{i},x\right)$3:**end for**   Sort $d_{i}\left(1,\ldots ,N\right)$ in ascending order Select the *K* cases $D_{x}^{K}$ closet to *x*Assign *x* to the most frequent class in $D_{x}^{K}$

A numerical measure that tells us how far one point is from another is known as distance. Some of the distances commonly used with the k-NN algorithm are given by expressions (19) to (21).

The Euclidean distance between points *x* and *y* is defined as:

(19) $$\begin{array}{cc} d_{e}\left(x,y\right)=d_{e}\left(y,x\right) & =\sqrt{\left({\left(y_{1},x_{1}\right)}^{2}+{\left(y_{2},x_{2}\right)}^{2}+\ldots +{\left(y_{n},x_{n}\right)}^{2}\right)}\\ & =\sqrt{\sum_{i=1}^{n}{\left(y_{i},x_{i}\right)}^{2}} \end{array}$$

The Chebyshev distance between two vectors of features *x* and *y* is the greatest of their differences along all their coordinate dimensions. This distance is defined by:

(20) $$d_{c}\left(x,y\right)=\max_{i}\left(\left|x_{i}-y_{i}\right|\right)$$

The Manhattan distance between points *x* and *y* of the n-dimensional feature space is the sum of the absolute differences in their coordinates:

(21) $$d_{m}\left(x,y\right)=\sum_{i=1}^{n}\left|x_{i}-y_{i}\right|$$

### 2.6. Cancer Datasets for Machine Learning

Three cancer datasets—Breast Cancer, Cervical Cancer, and Primary Tumour—were selected for preprocessing and analysis stages. These datasets have been used in similar studies based on machine learning techniques, and as can be seen in Table 2, the number of features, classes, and instances are different between each of these. The *Class distribution* column shows how unbalanced each one of the datasets is: the majority class of the Breast Cancer dataset is 2.4 times the minority class; in the Cervical Cancer dataset, the majority class is 14.6 times the minority class; and in the Primary Tumour dataset, this trend is only exacerbated when we compare the sixth class, which only has one instance versus the 338 instances of the other combined classes.

#### 2.6.1. Breast Cancer Dataset

The Breast Cancer dataset has been recently used to test the performance of artificial neural networks reported in literature [21]. The dataset contains information about the location, size, evolution time, and tumour type of a group of patients. The analysis of this representative sample allows common points in all these cases to be detected and allows specific points for particular cases to be identified. Specifically, the dataset includes 286 instances characterised by 9 features and 2 classes in which instances are classified.

#### 2.6.2. Cervical Cancer Dataset

The Cervical Cancer dataset—provided by Hospital Universitario de Caracas, Caracas, Venezuela—has been used in pattern recognition models [22]. This dataset comprises 858 instances characterised by 36 features and 2 classes in which instances are classified. Features include information about smoking, sexual history, use of intrauterine devices, sexually transmitted diseases, and diagnostic tests. This dataset needed special considerations in the preprocessing stage given that there are many missing values, since several patients had privacy concerns; additionally, this dataset contains four binary-class features that show the medical results of different tests: Hinselmann, Schiller, cytology, and biopsy. Only the latter was considered in this work.

#### 2.6.3. Primary Tumour Dataset

The Primary Tumour dataset has also been recently used to test the ability of artificial neural networks in multiclass classification tasks, as reported in [23]. This dataset includes 339 instances characterised by 17 features and 22 classes in which instances are classified. Features include information about the patient’s data such as age and sex, the histological characteristics of the tumour, and the list of the different organs that may or may not be affected by the tumour.

### 2.7. Methodological Approach Based on Data Mining

The exploration, preparation and modelling of the cancer datasets was carried out following the methodological approach provided by CRISP-DM (Cross Industry Standard Process for Data Mining), a widely proven method of guiding data mining work [24]. CRISP-DM covers six phases; the first two address the understanding of the domain’s problem and the available data. This then continues with the data preparation, the construction of the model, the evaluation of the proposed model and, finally, the presentation of the results.

Data preparation is one of the most critical phases in any methodological approach based on data mining. During this phase, the quality of the data is improved by cleaning, integrating, scaling, reducing dimensionality, transforming, and selecting relevant features from raw data, enhancing machine learning algorithms’ performance [25,26]. Data preparation can be seen as a phase that transforms real-world raw inconsistent and incomplete data into an understandable format. In this work, the data preparation phase was mainly based on the following activities:

- Replacing all missing values in the datasets with the mean (or mode);
- Normalising all numeric values in the dataset. The resulting values are in the range [0,1];
- Class balancing. Given the nature of the classification task, some class imbalance is expected;
- Producing *n* random subsamples of a dataset using sampling without replacement.

Regarding the modelling phase, the following considerations were taken:

- The same classifier (k-NN) was selected to achieve comparable results across all the experiments and to be able to evaluate patterns in the multi-class experiments;
- To obtain more reliable results from the pruned neural networks, we used this random validation methodology iteratively;
- A full RBM was used to obtain the classification baseline to determine if the pruning stage improved the error rate of the classification function;
- For all datasets, 8 different RBM configurations were implemented: the network’s input neurons depended on the dataset’s features, while the number of hidden units was [0.5,1,2,4,6,8,16,32] times the number of visible units.

## 3. Results

### 3.1. Cancer Dataset Preprocessing

As a result of the data preprocessing stage for cancer datasets, the following activities were carried out:

- All missing numeric, nominal, and ordinal feature values were replaced with the mean, mode, and median, respectively;
- All numeric values in each dataset were normalised; the resulting values are in the range [0,1];
- Class balancing was enhanced, improving the disproportionate ratio of observations in each class;
- The redundant features in the datasets were removed;
- The dataset was split into train and test subsets via 10-fold cross-validation.

**Figure 5:**
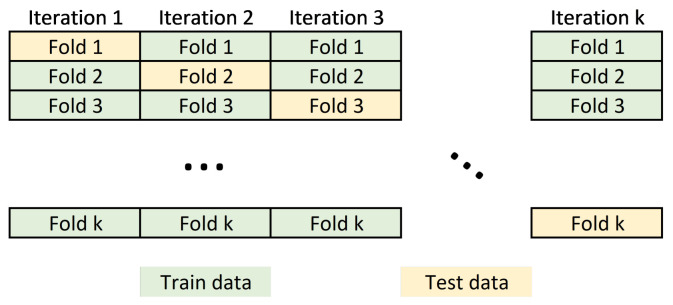
Cross-validation schema. In the *k*-fold cross-validation, the training set is split into k smaller sets. A model is trained using k−1 of the folds as training data; the resulting model is validated on the test set to compute a performance measure. The performance measure reported by *k*-fold cross-validation is then the average of the values computed in the loop.

The input dataset to the machine learning model usually requires partitioning the data into training and test sets. Data belonging to the training set contains a known output or label, from which the model learns to generalize to other data. On the other hand, the test set is used to test our model’s prediction capabilities. In this work, we perform a cross validation schema to split the dataset by partitioning the available data into three sets (see Figure 5).

### 3.2. Robustness Assessment of the Proposed Machine Learning Model

**Figure 6:**
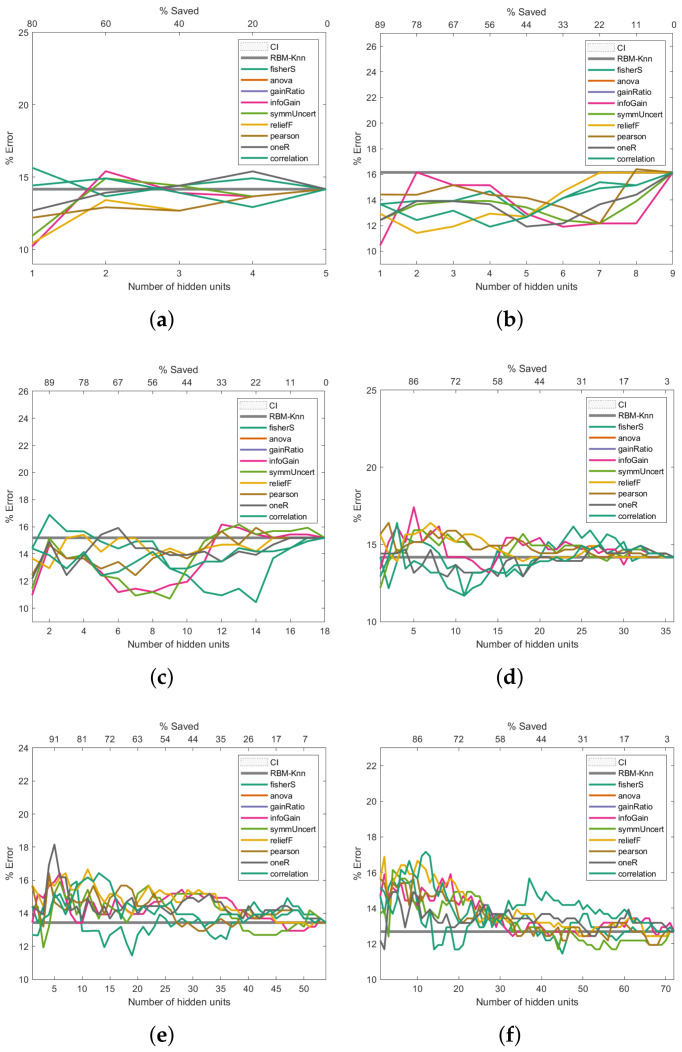

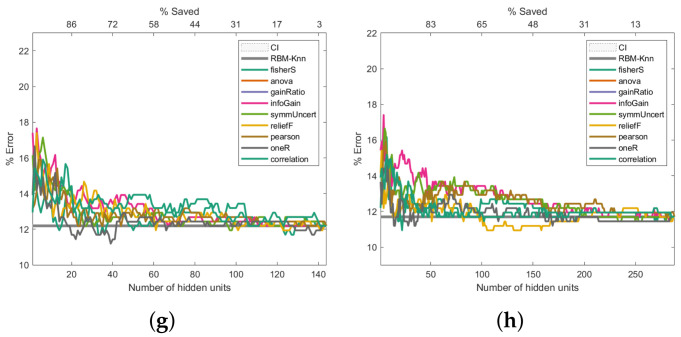
Pruning performance on the Breast Cancer dataset. Experiments were carried out with 5 (**a**), 9 (**b**), 18 (**c**), 36 (**d**), 54 (**e**), 72 (**f**), 144 (**g**) and 288 (**h**) initial hidden units.

**Figure 7:**
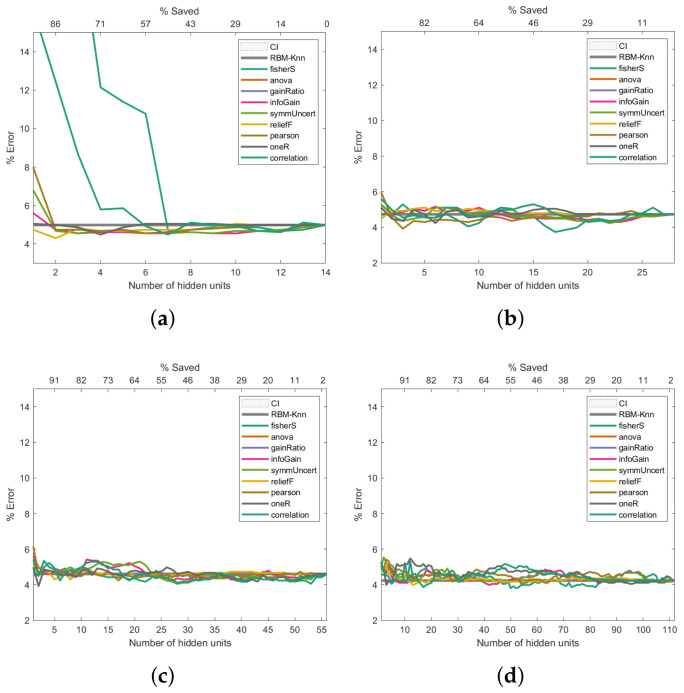

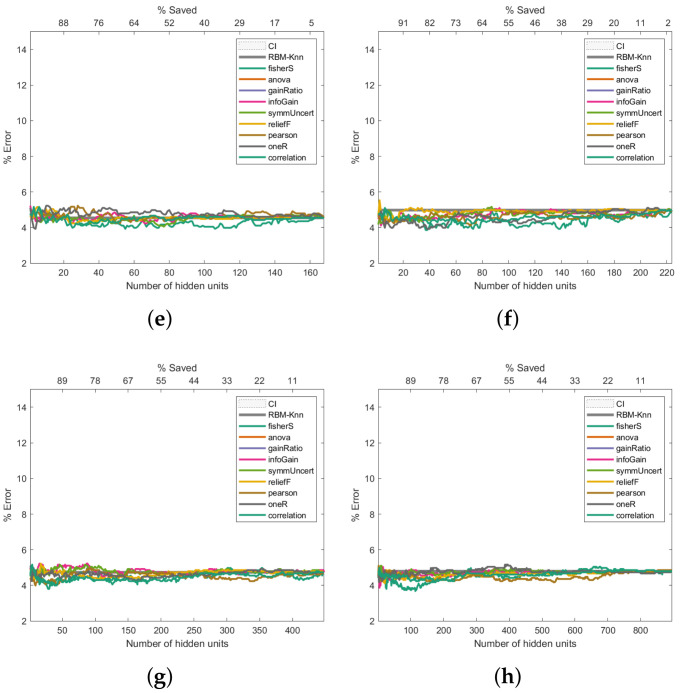
Pruning performance on the Cervical Cancer dataset. Experiments were carried out with 14 (**a**), 28 (**b**), 56 (**c**), 112 (**d**), 168 (**e**), 224 (**f**), 448 (**g**) and 896 (**h**) initial hidden units.

**Figure 8:**
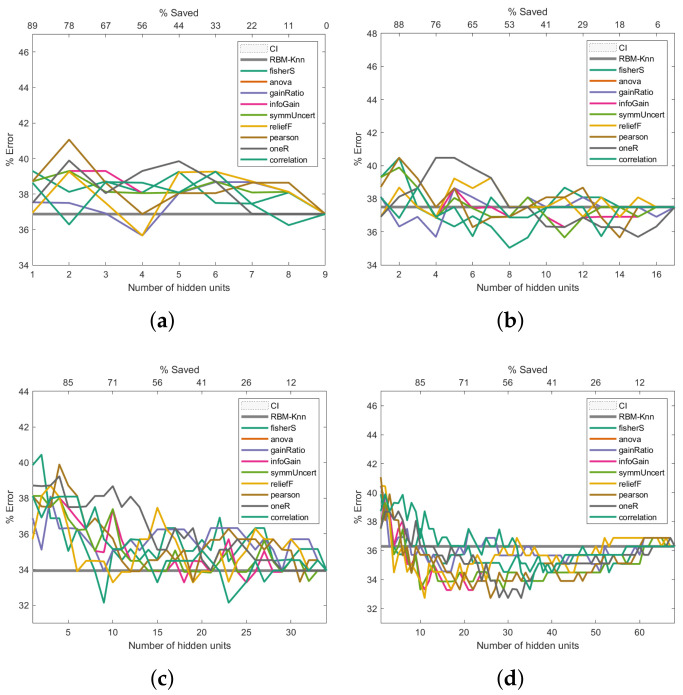

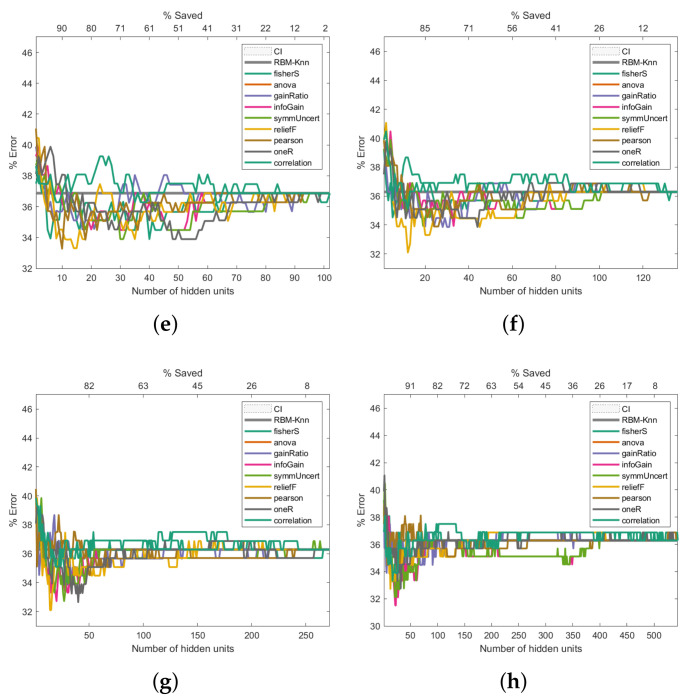
Pruning performance on the Primary Tumour dataset. Experiments were carried out with 9 (**a**), 17 (**b**), 34 (**c**), 68 (**d**), 102 (**e**), 136 (**f**), 272 (**g**) and 544 (**h**) initial hidden units.

To address how the proposed machine learning model could improve the error rate during the pattern recognition task, we assessed this model in three of the four cancer databases described in Section 2.6. For each of the information-entropy measures mentioned in Section 2.3, the classification error rates are shown in Figure 6, Figure 7 and Figure 8. The expression (22) was used to calculate the confidence interval with a confidence level of 95 per cent to assess if the classification errors are close to the baseline.

(22) $$CI=\bar{x}\pm z\frac{s}{\sqrt{n}}$$

Here, the sample mean is denoted by $\bar{x}$, the chosen z-value is denoted by *z*, the sample standard deviation is represented by *s*, and the sample size is indicated by *n*.

The same k-NN classifier was used to obtain comparable results across all experiments. The results presented in Figure 6, Figure 7 and Figure 8 are the average outcome of ten experiments randomly initialised. This repeated random validation scheme allowed us to obtain more solid and reliable results, which in turn permitted us to follow the pruned units’ error rate tendencies. Eight different RBM topologies were built for both databases where the number of neurons in the network’s input layer depended on the number of the dataset’s features, while the number of hidden units was [0.5,1,2,4,6,8,16,32] times the number of visible units. Table 3 shows the RBMs’ architectures.

### 3.3. Performance Analysis on the Breast Cancer Dataset

Figure 6 presents the Breast Cancer dataset experiments. The first experiment, shown in Figure 6a, reveals that the pruned RBM configurations with fewer hidden units than the number of features in the input samples improved the baseline error. The experiment presented in Figure 6b also shows that fewer hidden units than the number of input features produce better-than-baseline results. Although the results shown in Figure 6c,d are reasonably good, there is an increase in the error rates compared to the baseline. The trend of worsened error rates shown in Figure 6e–h matches the point where the number of hidden units surpasses the number of features in the input vectors, which not only does not improve the error rate but worsens it.

### 3.4. Performance Analysis on the Cervical Cancer Dataset

The experiments presented in Figure 7 use the Cervical Cancer dataset. In contrast to the experiments illustrated in Figure 6, the first experiment (Figure 7a) shows that any of the pruned networks did not improve the baseline. The second experiment (Figure 7b) shows that using 28 hidden units (same number of hidden neurons and input features) is more beneficial, since the general behaviour is improved. The result trend shown in Figure 7c–h, despite some being better-than-baseline, does not offer an evident error rate improvement. This behaviour is intriguing because it implies a point of diminishing returns in terms of the error rate versus the number of hidden units. We hypothesise that the best this proposal can do for this dataset is around a 4% error rate, which is why adding more hidden units is worse.

### 3.5. Performance Analysis on the Primary Tumour Dataset

Figure 8 shows the Primary Tumour experiments. These exhibit a similar trend to the Breast Cancer results: the higher the number of hidden units, the better the error rate (Figure 8c–h). However, unlike the Breast Cancer results (Figure 6), the improvement in the error rate is not as noticeable; even 544 hidden units were insufficient to improve the classification past 30%. Class imbalances, the fact that there is missing data, or the complexity of the dataset could be factors that contribute to the high error rate.

### 3.6. Summarized Results

As shown in Figure 6, Figure 7 and Figure 8, the Fisher score and reliefF are the most robust measures to achieve the best error rate with the shallowest network. We will initially refer to the analysis carried out on the Breast Cancer dataset to exemplify this.

As shown in Figure 6a, the pruning performed by reliefF produces an error close to 11% when the number of hidden neurons is equal to 2 (which means a saving of 78% with respect to the 9 initial hidden neurons). In contrast, the pruning performed by Fisher Score leads to an error slightly higher than the first one, but below 12% when the number of hidden neurons is equal to 4 (which means a saving of 56% with respect to the initial number of hidden neurons). On the other hand, as can be seen in Figure 6c,d, the pruning that led to the most minor error is performed by the Fisher score; when the initial number of hidden neurons is equal to 18 (Figure 6c), the Fisher score produces an error close to 10.5% and a saving of 22%, while an error below 12% and a saving of 72% are reached when the total number of hidden neurons was set at 35 (see Figure 6d).

The Fisher score was again the most robust measure to achieve the best error rate with the shallowest network regarding the analysis performed on the Cervical Cancer dataset. As shown in Figure 7b, when the initial number of hidden neurons is equal to 28, the Fisher score carried out the pruning that led to the least error, close to 3%, with savings above 40%. Note in Figure 7c–h that again, the Fisher score produces the least error. In these cases, although some results are better than the baseline, the overall performance does not offer a noticeable improvement in the error rate. In particular, when starting from an initial number of 55 or 110 hidden neurons (Figure 7c,d), reliefF offers outstanding results, saving more than 80% of hidden neurons.

Finally, regarding the Primary Tumour Dataset, the Fisher score and reliefF follow the same trend as that observed for the two previous datasets; these 2 measures produce the lowest error rates when the initial number of hidden neurons are 16 and 30, respectively, as can be seen in Figure 8b,c. The Fisher score measure is the one that produces the lowest error rate, with a saving of hidden neurons that ranges between 60% and 75%. However, it should be noted that for an initial number of hidden neurons greater than or equal to 60 (Figure 8d–h), reliefF leads to a better error rate than the Fisher score, with hidden neurons savings over 90%.

### 3.7. Strengths and Weaknesses

The post-training pruning (PTP) approaches begin with training a big enough artificial neural network to meet the desired performance level. Following that, neurons are eliminated from the trained network, which is then fine-tuned or retrained. This operation, however, is carried out without taking into account the pattern’s class information, i.e., it is unsupervised, and there is no straightforward method to assess the influence of weight pruning on class categorisation.

We addressed two shortcomings of the PTP techniques in this work: (1) the retraining phase’s computing costs, and (2) the pruning process’ influence on supervised multiclass classification. Our suggested method is similar to feature selection in that it ranks neurons and removes those with poor discriminative abilities and weights, allowing for a more accurate assessment of the pruning process. Another advantage of this method over traditional methods is that it does not require additional training after pruning.

On the other hand, it should be emphasised that the proposed pruning strategy has room for improvement. One of the essential issues is determining which information-entropy-based metric delivers the most significant results for distinguishing across classes. We observed that two of these information-entropy-based metrics (Fisher score and reliefF) provided superior outcomes, but we cannot say which one is the best because the only parameter we have to compare them with is the error % in the classification of the various cancer datasets. Given that we analysed the influence of a subset of discriminative measures, the results obtained are inherently local to this subset.

## 4. Summary and Conclusions

The cancer datasets analysed in this work have been used in the past and, although the objective of our proposal was not to obtain the best error rate but to investigate the post-training neuronal pruning methodology using dissimilarity measures inspired in the information-entropy theory, the results obtained after pruning up to 90% of the neural network were favourable. As shown in Table 4, the reported results for the Breast Cancer dataset is 22% error rate while our findings range from 10% to 15%; for the Cervical Cancer dataset, the reported best error rate is 31% while our proposal error rates are in the range of 4% to 6%; and for the Primary Tumour dataset the reported error rate is 42%, and our best error rate is 31%. Given these results, the following inferences can be derived:

- Favourable trends were achieved with savings of 60 to 95 per cent of hidden neurons. This suggests that the implementation of the ranking strategy is quite favourable for the pruning process discussed in this work;
- The two most robust ways to rank and prune the neurons to achieve a reasonable error rate with the shallowest network are the Fisher score and reliefF;
- The findings in this work suggest that it is better to prune a bigger network than to find a shallow neural network empirically.

**Table 4:** Reported best error rate vs. best error found with the proposed pruning method for the cancer datasets under analysis.

Cancer Dataset	Reported Error Rate	Error Rate Found with the Proposed Pruning Method
Breast Cancer	(ANN) 10.68% [21]	10% to 15%
Cervical Cancer	(Linear Regression) 31% [22]	4% to 6%
Primary Tumour	(ANN) 20.35% [23]	31%

Post-training pruning (PTP) methods begin the training process with the largest neural network capable of executing the classification task with the expected performance level. These methods start with a large enough neural network to contain an extensive number of hidden neurons. Afterwards, after the least relevant neurons for the classification task are pruned, the neural network is retrained. Two PTP methods [27,28] were used to search for the optimal neural network topology; both delete the less-relevant neural connections. The pruning process is unsupervised in PTP methods, i.e., the class information of each pattern is not taken into account. Therefore, there is no straightforward way of determining the effect of pruning weights on the classification task.

PTP has some restrictions that we addressed; the first relates to the retraining process’ computational costs for PTP methods. For the pruning method proposed here, a further retraining phase is not required. The second restriction of the PTP methods lies in the fact that the pruning process is carried out in an unsupervised way. To deal with this restriction, in our pruning method, the class information of each pattern is taken into account. The pruning method presented here ranks neurons according to their discriminative capabilities, resembling a feature selection.

One parameter in the methodology needs further investigation: a discriminatory feature that offers the best result for inter- and intra-class separability. To this end, we have used a collection of discriminatory measures widely used for pattern recognition. Nonetheless, as pointed out in the Summary discussion, two of these measures (ReliefF and the Fisher score) have achieved better results. It is hard to prove any of these to be the best overall measure since the set of discriminatory measures has been chosen a priori, and any result obtained would be inherently local to that dataset. Additionally, only the classification performance of these metrics on the various datasets was assessed. Despite this, the herein proposed dissimilarity-based PTP method achieved a reduction of up to 90% from the original neural network size, making it more efficient in terms of time, memory, and the number of computations while maintaining its accuracy.

To summarise, in this work, we explored the effect of a novel pruning model. To this end, we used an RBM and a method for pruning the less salient neurons in medical data processing for cancer research. Even though an RBM was used, our approach may also be applied to other types of neural networks, which could be characterised by a significant computation and parameter storage increase, such as convolutional neural networks (CNNs). The proposed model helped identify the role of a group of information-entropy measures to discover the most diverging neurons in medical data analysis. As described by [5], our approach adds class information to the pruning algorithm, which can be interpreted as a feature extraction process applied to the hidden neurons. We decided on datasets from the medical cancer domain, specifically the Breast Cancer, Cervical Cancer, and Primary Tumour datasets, to test this model.

As our results show, the preprocessing stage is an essential step in medical data analysis because the success of machine learning techniques depends mostly on proper data filtering. As mentioned before, the quality of medical data is considerably improved during the preprocessing phase by applying techniques such as cleaning, integration, normalisation, size reduction, and the extraction of relevant features. However, we have to keep in mind that even after having the best medical data quality at the preprocessing stage, there could be problems in the dataset. Some data may be incorrect or missing after the processing stage.

Scientific research continually generates information, and frequently, the analysis of the generated data is practically impossible due to its large volume, variety, and veracity. Therefore, it is necessary to discriminate information. In this sense, preprocessing, information-entropy measures, and analysis methods play a crucial role since they allow the most critical elements to be selected in a significant way, which is very helpful for the creation of computational tools as a support for the timely diagnosis of cancer.

## Figures and Tables

**Table 1:** Definitions.

Term	Definition
Artificial neural network (ANN)	Artificial neural networks are information processing systems inspired by biological neural networks. These processing systems are designed to emulate a series of functions carried out by the brain, such as classification, prediction, pattern recognition, learning, and generalising knowledge.
Artificial neuron	An artificial neuron is a mathematical function that models the biological neuron. It receives weighted inputs x and produces an output y, typically calculated by a non-linear function of the inputs.
Visible neuron/unit	The visible units or neurons make up the input layer in a multilayer artificial neural network. Those neurons receive the information that the neural network will process.
Hidden neuron/unit	The hidden units or neurons make up the intermediate layers in a multilayer artificial neural network. Those neurons, located between the input and output layers, process the input data.
Network topology	The network topology refers to the number of layers, the number of neurons per layer, and the connection pattern established within and between layers.
Size of the network	The size of an artificial neural network is related to the total number of neurons and layers that make it up.
Pruning method	The technique used to reduce the size of a neural network to make it more efficient in terms of resources such as execution time, memory usage, energy, and the number of computations required.
Restricted Boltzmann machine (RBM)	A RBM is a neural network that models a non- directed and symmetrical connection between the visible and hidden nodes without any intra-layer connection.
Deep neural network	A deep neural network uses a hierarchy of intermediate representations (hidden layers) to learn high-level data representations. A deep neural network has several layers of hidden neurons between the input and output layers.
Deep belief network (DBN)	A DBN can be seen as a deep neural network where each layer typically comprises several RBMs. A DBN learns the internal representation of the input data and can also learn to reconstruct its inputs.
k Nearest Neighbor (k-NN)	The k-NN algorithm can be defined as memory- based learning or instance-based learning, in which the model is created by memorising the training data set and then using this data to make predictions.

**Table 2:** Cancer datasets.

Cancer Dataset	# of Original Features	# of Instances	# of Classes	Class Distribution
Breast Cancer	9	286	2	[201, 85]
Cervical Cancer	35	858	2	[803, 55]
Primary Tumour	17	339	21	[84, 20, 9, 14, 39, 1, 14, 6, 2, 28, 16, 7, 24, 2, 1, 10, 29, 6, 2, 1, 24]

**Table 3:** Architecture of the restricted Boltzmann machines.

Cancer Dataset	# of Features	# Test Hidden Layer Architectures
Breast Cancer	9	[5, 9, 18, 36, 54, 72, 144, 288]
Cervical Cancer	28	[14, 28, 56, 112, 168, 224, 448, 896]
Primary Tumour	17	[9, 17, 34, 68, 102, 136, 272, 544]

## Data Availability

The Breast Cancer dataset that supported the findings of this study is available in the UCI Machine Learning Repository, https://archive.ics.uci.edu/ml/datasets/Breast+Cancer, accessed on 24 November 2021. The Cervical Cancer dataset that supported the findings of this study is available in the UCI Machine Learning Repository, https://archive.ics.uci.edu/ml/datasets/Cervical+cancer+(Risk+Factors), accessed on 24 November 2021. The Primary Tumour dataset that support the findings of this study are available in the UCI Machine Learning Repository, https://archive.ics.uci.edu/ml/datasets/primary+tumor, accessed on 24 November 2021.
